# Correction: Poly-IC enhances the effectiveness of cancer immunotherapy by promoting T cell tumor infiltration

**DOI:** 10.1136/jitc-2020-001224corr1

**Published:** 2024-04-04

Sultan H, Wu J, Fesenkova VI, *et al*. Poly-IC enhances the effectiveness of cancer immunotherapy by promoting T cell tumor infiltration. *J ImmunoTher Cancer* 2020;8:e001224. doi: 10.1136/jitc-2020-001224

Figure 5D and the associated caption has been updated due to the data being inserted incorrectly in the originally supplied figure.

